# Stanniocalcin-1 in tumor immunity: acts via macrophages

**DOI:** 10.3389/fimmu.2024.1510182

**Published:** 2024-11-25

**Authors:** Lele Wang, Jianjun Wang, Weijie Qiang, Weihong Ge

**Affiliations:** ^1^ Department of Pharmacy, Nanjing Drum Tower Hospital, School of Basic Medicine and Clinical Pharmacy, China Pharmaceutical University, Nanjing, Jiangsu, China; ^2^ School of Basic Medicine and Clinical Pharmacy, China Pharmaceutical University, Nanjing, Jiangsu, China; ^3^ Department of Pharmacy, Nanjing Drum Tower Hospital, Affiliated Hospital of Medical School, Nanjing University, Nanjing, Jiangsu, China

**Keywords:** tumor immunity, tumor-associated macrophages, Stanniocalcin-1, immunotherapy, tumor microenvironment

## Abstract

Tumor immune escape has become a research hotspot in the field of cancer immunotherapy. Tumor-associated macrophages (TAMs) are the key component of tumor microenvironment, which play a pivotal role in tumor immune escape by regulating the immunity checkpoints, inhibiting the activity of T lymphocytes and natural killer (NK) cells, and modulating proportion of different T cells. Stanniocalcin-1(STC1)is ubiquitously expressed in human body, which is proven to involve with tumor progression and clinical prognosis. Recently, STC1 is implicated in tumor microenvironment as a phagocytosis checkpoint, as well as regulates the immunity via macrophages. In the review, we discussed the role of STC1 and TAMs in tumor immunity and their crosstalk, hoping to provide references for the research of STC1 in tumor immunotherapy.

## 1 Introduction

Tumor is one of the leading causes of death worldwide. In 2022, there were an estimated 20 million cases of cancer diagnosed and 9.7 million deaths due to cancer globally, which severely threatens human life and health (1). Traditional treatments for cancer include surgery, radiotherapy, and chemotherapy. However, these options, alone or in combination, are not effective and there is a pressing need for therapeutic progress. In recent years, molecular targeted therapy and immunotherapy have gradually attracted researchers’ interest in the field of oncology. Unfortunately, in clinical practice, only approximately 20%-40% of patients could benefit from immunotherapy, and the majority of patients would appear drug resistance and adverse drug reactions (2). “Tumor immune escape” is considered as the main reason for the immunotherapy failure, tumor cells evade the attack of immune system by decreasing tumor immunogenicity, regulating the infiltration of immune cells, interfering with immune checkpoints, destroying the recognition function of immune cells, recruiting immunosuppressive cells, etc (3). Accordingly, the research and identification of underlying genes and molecules involved in tumor immune escape is of great significance to decrease drug resistance and improve the efficacy of immunotherapy.

Tumor progression is the crosstalk between cancer cells and their living environment–the tumor microenvironment (TME), which consists of immune cells, blood vessels, fibroblasts, and mesenchymal stromal cells, etc (4). Hence, TME greatly influences the efficacy of immunotherapy (5). As the key component of TME, macrophages are recruited around solid tumors via tumor cell-derived chemokines, thus are named as tumor-associated macrophages (TAMs) (6). It has been found that high TAMs infiltration in tumors is typically associated with poor prognosis and tumor progression, suggesting that TAMs correlate with tumorigenesis and development (7, 8). Reportedly, TAMs can be involved in tumor immune escape by regulating PD-1/PD-L1 axis (9, 10), inhibiting the activity of T lymphocytes and natural killer (NK) cells (11), as well as modulating the conversion of T cells into regulatory T cells (Tregs) (12). In conclusion, TAMs play a pivotal role in tumor immune surveillance and immune escape, so the discovery of underlying molecular mechanisms regulating TAMs function is of great significance for tumor immunotherapy strategies.

Stanniocalcin-1 (STC-1), a glycoprotein hormone first identified in bony fish, relatives to calcium and phosphorus metabolism (13). STC1 is ubiquitously expressed in human body, encompassing the brain, thyroid, spleen, thymus, parathyroid glands, lungs, heart, and skeletal muscle, and acts through autocrine and paracrine (14). So far, multiple experimental studies have demonstrated that STC1 affected cancer cell proliferation (15), migration (16), metastasis (17), apoptosis (18), stem cell properties (19), epithelial-mesenchymal transition (EMT) (20), etc. Moreover, a number of clinical studies have validated that STC1 is aberrantly expressed in diverse tumor tissues and associated with patients prognosis (13, 21, 22). In addition, STC1 is involved in many cancer-related signaling pathways, such as notch1-sex determining region Y-box 2 (Notch1-SOX2) (23), extracellular signal-regulated kinases1/2 (ERK1/2) (24), nuclear factor-kappaB (NF-ΚB) (17) and jun-N-terminal kinase (JNK) (25) signaling pathways, etc. In brief, STC1 is associated with multiple physiological and pathophysiological processes of tumorigenesis and progression.

In recent years, some studies have found that STC1 is implicated in tumor microenvironment as a phagocytosis checkpoint. In detail, STC1 participates in tumor immunity by affecting the TME, participating in the EMT, and interfering the phagocytic signals. Moreover, multiple researches have demonstrated that STC1 serves as a regulator of tumor immunity affecting differentiation, activation, inflammatory response, and antigen presentation of macrophages. In the review, we introduced biological function and expression pattern of STC1, discussed the role of STC1 and TAMs in tumor immunity, explored how STC1 regulates the function of macrophages. In addition, we proposed the future development directions and prospects for immunotherapy, hoping to provide references for the research of STC1 in tumor immunotherapy.

## 2 Biological function and expression pattern of STC1

### 2.1 Structure and molecular characteristics of STC1

The STC family consists of STC1 and STC2, and STC1 is a glycoprotein hormone that was first discovered in bony fish. In 1996, STC1 is shown to exist in mammals and there is 73% homology of STC1 amino acid sequences between human and bony fish (26). In human, the STC1 gene is consisted with four exons with a 5’UTR rich in CAG trinucleotide repeats (27) and locates on the short arm of chromosome 8p11.2-p21. Meanwhile, STC1 protein is a homologous dimer glycoprotein containing 11 conserved cysteine residues, composed of 247 amino acids, with a molecular weight of 56 kDa (28). STC1 is ubiquitously distributed in brain, thyroid, spleen, thymus, parathyroid gland, lung, heart, skeletal muscle, kidney, pancreas, small intestine, colon, placenta, ovary, testis and prostate, etc (14). The widespread expression of STC1 indicates that it acts through autocrine and paracrine. STC1 is considered as a regulator of calcium and phosphorus metabolism and associated with tumor progression and metastasis (29, 30). What’s more, its localization in the thymus and spleen reveals that it may make a difference in immune and inflammatory processes (10). STC1 is secreted to extracellular environment *in vivo* (31), and binds to protein on the cell membrane (32), followed by internalization and targeting to the inner mitochondrial membrane (33, 34). There is no clear consensus on the receptor of STC1 at present, but STC1 can play a regulatory role in the occurrence and development of cancer through signaling pathways such as Notch1-SOX2 (23), ERK1/2 (24), NF-Κb (17) and JNK (25) signaling pathways, etc.

### 2.2 Expression of STC1 in tumor cells

Emerging evidences have demonstrated that STC1 is abnormally expressed in tumor tissues and is associated with tumor cell proliferation, migration, apoptosis and disease prognosis, such as non-small cell lung cancer, renal cell carcinoma, leukemia, lung adenocarcinoma, glioma, colon cancer, prostate cancer, ovarian cancer, etc.

A meta-analysis involving 16 articles and 2,942 participants found that high expression of STC1 was significantly associated with poor prognosis, especially for digestive (HR: 1.73; 95% CI: 1.36–2.20) and nervous system tumors (HR: 2.60; 95% CI: 1.29–2.25) (35). Chromosome 8p deficiency disorder usually appears in colorectal cancer. By analyzing gene expression on chromosome 8p in 44 patients, a total of 13 genes located in 8p21-22 2Mb region were identified, including STC1, may trigger the progression and metastasis of colorectal cancer. In ovarian cancer, Liu et al. (36) found that the expression level of STC1 protein increased. Silencing STC1 expression by siRNA showed that STC1 could promote cell proliferation, migration and colony formation. What’s more, STC1 could increase the growth of xenograft tumors. Mechanically, this may be because STC1 could increase the expression of cell cycle proteins (cyclin A, cyclin B1, cyclin-dependent kinase 2, and a short cyclin E isoform) to promote G1 into S phase; and upregulate antiapoptotic proteins (B-cell lymphoma 2, B-cell lymphoma-extra large) expression. Consistent with ovarian cancer, in lung adenocarcinoma (15) and clear renal cell carcinoma (20), high expression of STC1 had also been shown to regulate the levels of cell cycle proteins and apoptotic proteins, thereby facilitating cell proliferation. However, in breast cancer, the expression and prognostic value of STC1 are different in distinct subtypes. In triple negative breast cancer (TNBC), the expression level of STC1 is higher than that in normal tissues, and is associated with poor prognosis (17). In hormone receptor-positive breast cancer, high expression of STC1 is associated with good prognosis (28). In human epidermal growth factor receptor 2^+^ (HER2^+^) breast cancer, high STC1 expression was not associated with prognosis (37).

In summary, the crosstalk between STC1 expression and prognosis is different across diverse tumor types (**Table 1**). Usually, STC1 is highly expressed in most tumors and is associated with poor prognosis. However, in some highly heterogeneous tumors, such as breast cancer, STC1 may be differentially expressed in different tumor subtypes and sizes, which may be affected by the copy number variations, gene expression profile of different tumor subtypes. Moreover, some studies have indicated that in gynecological malignancies, the loss of STC1 function may impact normal physiological functions, which possibly leads to the low expression of STC1 in tumor tissues. And the expression level of STC1 fluctuates throughout the entire menstrual cycle of women, the changes in calcium metabolism at different age stages in women also affect the level of STC1 (38). In the future, more clinical samples and comprehensive analysis are needed to explore the expression of STC1 in multiple tumor tissues in well-stratified disease groups. In addition, clarifying the mechanism and causes of differential expression of STC1 will help us understand the role and value of STC1 in tumor immunity.

**Table 1:** Expression and function of STC1 in tumors.

Tumor type		Detected methods	Sample sizes	Expression	Function	References
Prostate cancer		IHC	22	High expression	Promote growth and metastasis	(80)
Colorectal cancer		qRT-PCR	20	High expression	Promotes migration and invasion, associated with poor postoperative prognosis	(16)
Renal cell carcinoma		qRT-PCR, WB, IHC	48	High expression	Promotes growth, proliferation and EMT, associated with Fuhrman tumor grade and TNM stage	(20)
Laryngeal squamous cell carcinoma		qRT-PCR	62	High expression	Associated with advanced clinical stage	(81)
Ovarian cancer		IHC	342	High expression	Increase cell adhesion, invision, proliferation and migration, colony formation, and promote the growth of transplanted tumor	(40, 82)
Lung cancer	Non-small cell lung cancer	qRT-PCR	65	High expression	Associated with advanced clinical stage and histological subtype	(83)
	Lung adenocarcinoma	ELISA, IHC	88	High expression	Increase cell proliferation, promote G1/S transition in the cell cycle, inhibit apoptosis	(15)
Breast cancer	ER^+^/TAM^+^	IHC	541	High expression	Associated with prognosis	(84)
	ER^-^/TAM^-^	IHC	300	Low expression	Associated with prognosis	(84)
	TNBC	qRT-PCR	cells	High expression	Associated with RFS、OS, promote metastasis	(17)
	All subtypes	IHC	137	High expression	Promote tumor growth and drug resistance	(85)
Hepatocellular carcinoma		qRT-PCR, IHC, WB, ELISA	125	High expression	Promote metastasis, promote stemness, and positively correlate with tumor size	(19, 25)
Cervical cancer		qRT-PCR, IHC	15	Low expression	Inhibit cell proliferation, migration and invasion	(86)
Leukemia		ELISA, IHC	20	High expression	Promotes chemotherapy resistance, a marker of MRD, associated with prognosis	(87)
Gastric cancer		IHC, ELISA	83	High expression	Associated with lymph node metastasis and advanced clinical stage	(88)
Glioma		qRT-PCR, IHC	80	High expression	Associated with high pathological grade	(89)
Thyroid cancer		IHC	100	High expression	Associated with cancer differentiation	(90)
Esophageal squamous cell carcinoma		qRT-PCR	15	High expression	Associated with advanced T stage and prognosis	(22)
Bladder cancer		IHC	63	High expression	Associated with the stage and poor prognosis of bladder cancer	(39)

EMT, epithelial-mesenchymal transition; ER, Estrogen receptor; TAM^+^, tamoxifen positively; TAM^-^, tamoxifen negatively; TNBC, triple negative breast cancer; RFS, relapse-free survival; OS, overall survival; MRD, minimal residual disease.

### 2.3 STC1 involved in tumor immunity

It is clear that STC1 regulates tumor immunity by influencing TME, participating in EMT and interfering with phagocytosis signals. By searching TCGA database, Sun et al. (39) found that STC1 was positively correlated with the expression of immune checkpoints, such as PD-L1 (k=0.29), PD-L2 (k=0.39), OX40L (k=0.32), TIM3 (k=0.31), FOXP3 (k=0.24), CTLA4 (k=0.23). Moreover, this study found that the high expression of STC1 was correlated with the high level of immune cells in TME, encompassing TAMs, CD4^+^ T cells, CD8^+^ T cells, and total T cells, indicating that STC1 was associated with immune checkpoints and multiple immune cells infiltration. That is, STC1 exhibits key functions in tumor immunity by regulating the level of immune cells in TME. STC1 has higher expression in cancer stroma and cancer associated fibroblasts (CAFs) than normal stroma and normal fibroblasts (NFs) (40). By detecting the levels of EMT markers fibronectin, vimentin and slug, it was found that human recombinant STC1 (rhSTC1) promoted EMT thus triggering the proliferation and metastasis of ovarian cancer cells via Akt phosphorylation. While monoclonal antibody STC1 (STC1 Ab) could reverse the above effects. By constructing STC1 over-expression cell to further explore its mechanism, it was found that STC1 secreted by cancer cell promoted the transformation from NFs into CAFs, but detailed mechanisms were not be clarified (40). In this way, STC1 participate in tumor immunity. In 2021, STC1 was reported to be involved in tumor immunity as a phagocytic checkpoint. Specifically, STC1 could damage the phagocytosis of antigen presenting cells (APC) and the activation of T cells by capturing calreticulin (CRT), thereby blocking the “eat me” signal of tumor cells and mediating tumor immune escape. And it was found that CRT interacted with STC1 through protein disulfide isomerase associated 3 (PDIA3) by STC1-FLAG immunoprecipitation (IP) (41). Using an *in vitro* cell co-culture model including CD19 antigen receptor T-cell immunotherapy (CAR-T) cells, tumors cells and macrophages, Zhang et al. found human mesenchymal stem cell (hMSCs) could suppress the efficacy of CD19 CAR-T therapy by reducing the proportion of CD8^+^ T cells, increasing the proportion of CD4^+^ T cells and Treg cells, and promoting IDO and PD-L1 levels, while STC1 knockdown could eliminate these effects. Compared with hMSCs^shctrl^, hMSCs^shSTC1^ significantly decreased the expression level of IDO and PD-L1 protein by more than 50%. Moreover, hMSC^shSTC1^ combined with CD19 CAR-T cells therapy showed a curative effect in a xenograft model with tumors closely disappeared at day 38. In a word, the stimulatory signals appearing in the process of CAR-T treatment enabled hMSCs to secrete STC1, and exerted immunosuppressor function (12). In summary, STC1 affects tumor immunity by regulating immune checkpoints, affecting phagocytosis signals, impacting T cell immune response, and participating in EMT.

## 3 Tumor-associated macrophages participate in tumor immune escape

### 3.1 TAMs and tumor immune escape

**Figure 1:**
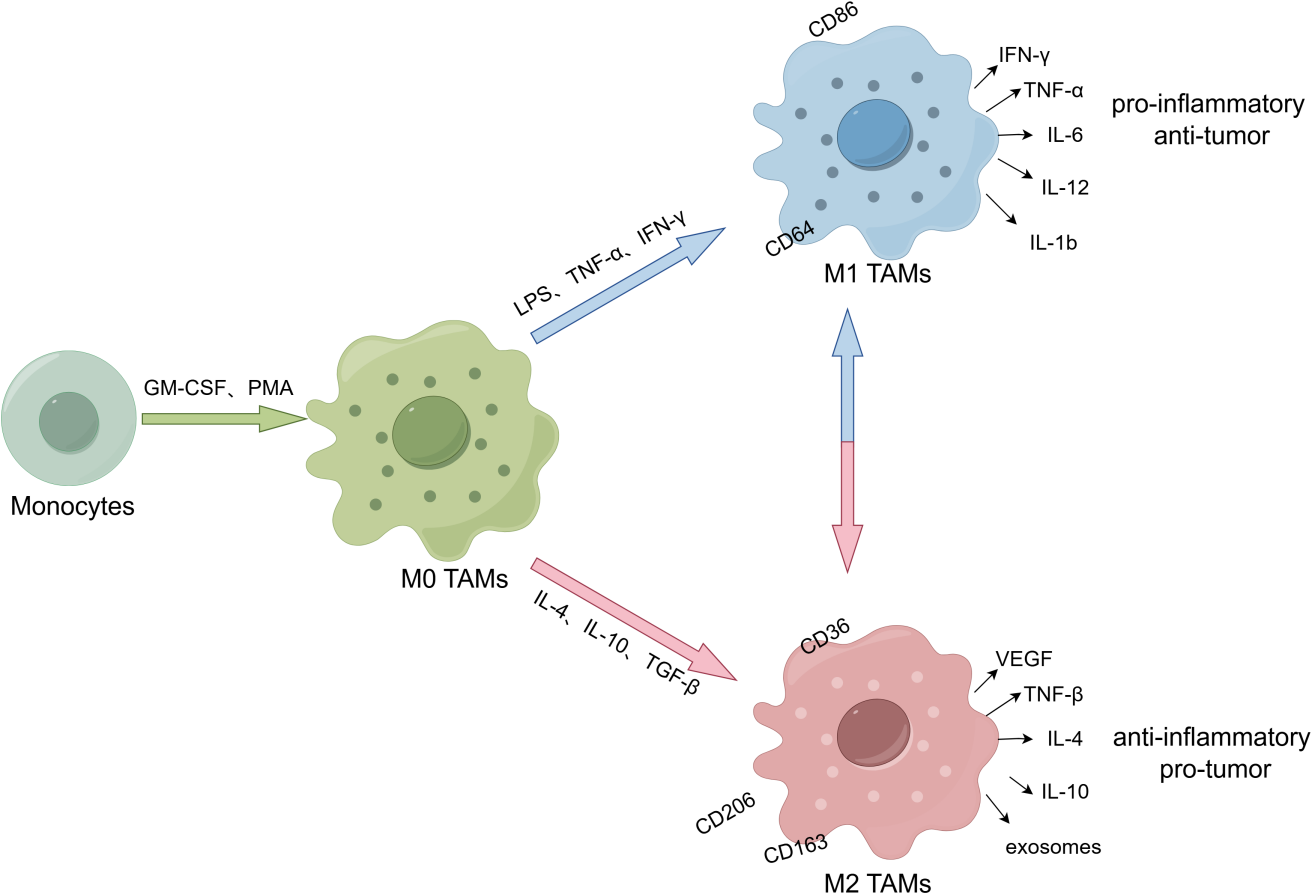
The polarization and subpopulation differentiation of macrophages. (By Figdraw.). M0 TAMs are induced from monocytes by granulocyte macrophage colony-stimulating factor (GM-CSF) and phorbol 12-myristate 13-acetate (PMA). M0 TAMs are stimulated and induced into different subtypes under cytokines. M1-type macrophages are stimulated by LPS, TNF-α and IFN-γ, and secrete pro-inflammatory cytokines (IL-6, IL-12, IL-1b and TNF-α) and chemokine ligands (CCL2, CCL5, CXCL9, CXCL10 and CXCL11). Its markers include CD86 and CD64, etc, which have pro-inflammatory and anti-tumor effects. While M2-type macrophages are stimulated by IL-4, IL-10 and TGF-Β, and secrete IL-4/10, TGF-Β, VEGF and exosomes. Its surface markers include CD36, CD206 and CD163, etc, which have anti-inflammatory and pro-tumor effects.

TAMs contains two subtypes, the M1 type of classical activation pathway and the M2 type of alternate activation pathway (42). As shown in **Figure 1**, M1-type macrophages are stimulated by lipopolysaccharide (LPS), tumor necrosis factor-α (TNF-α) and interferon-gamma (IFN-γ), and secrete pro-inflammatory cytokines (IL-6, IL-12, IL-1b and TNF-α) and chemokine ligands (CCL2, CCL5, CXCL9, CXCL10 and CXCL11), which have pro-inflammatory and anti-tumor effects. Its common markers are CD86, CD64, CD80, CD16, CD120b, TLR2 and STAT1. While M2-type macrophages are stimulated by IL-4, IL-10 and transforming growth factor-beta (TGF-Β), and secrete IL-4/10, TGF-Β, VEGF and exosomes. Its common surface markers are arginase, CD36, CD206, CD266 and CD163, which have anti-inflammatory and pro-tumor effects (43). What’s more, cellular heterogeneity and unique subtypes of TAMs have been widely discovered recently with the development of high-dimensional flow cytometry and transcriptomic profiling (44). Generally, TAMs are mainly M1-type in the early stage of tumor development, which exert phagocytic effects on tumor cells and exhibit anti-tumor function. However, under the influence of TME, M1-type TAMs are gradually polarized into M2-type TAMs, which exhibit immunosuppressive impacts and participate in the formation of tumor immunosuppressive microenvironment (45). Compared with the tumor center, M2-type TAMs infiltration at the tumor invasive margin increased and was associated with low survival rate, indicating that TAMs distribution also had a certain spatial heterogeneity (46). In addition, the distribution of TAMs varies among different types of tumors, possibly due to differences in tumor immunosuppressive status, aggressiveness, and gene expression profiles (47). In a word, the phenotype and function of TAMs may be significantly different across tumor types and different stages of tumor development. The time-space heterogeneity determines the specific role of TAM in TME to a large extent.

**Figure 2:**
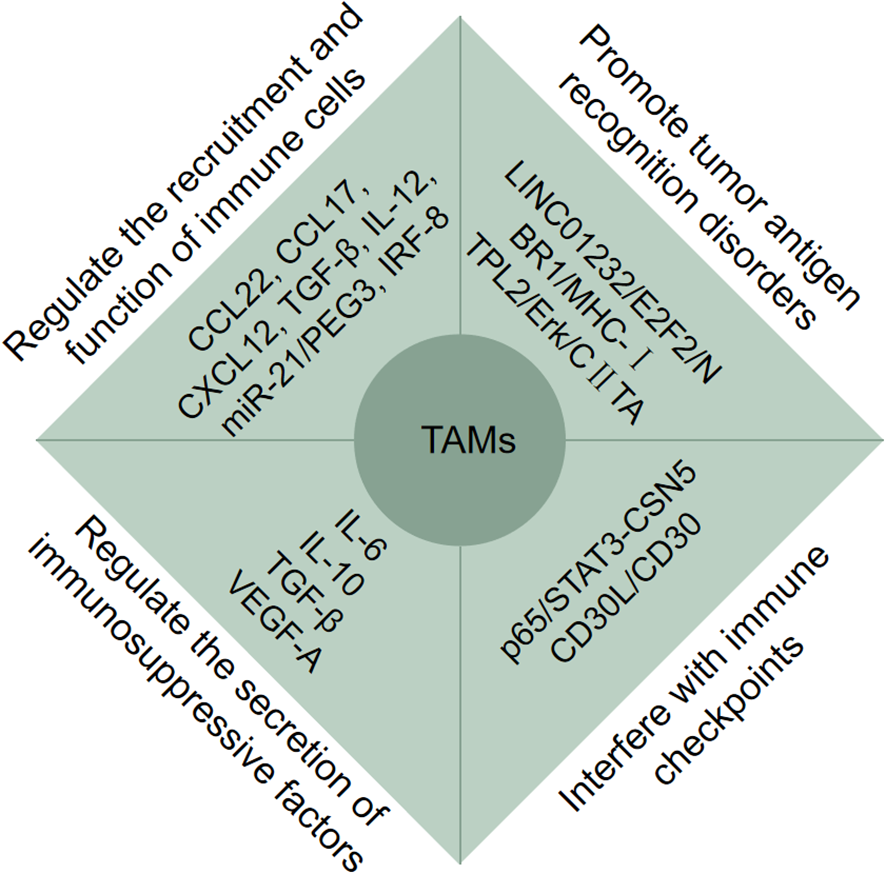
Tumor-associated macrophages (TAMs) participate in tumor immune escape. TAMs participate in tumor immune escape through multiple pathways. Firstly, TAMs regulate the recruitment and function of immune cells via CCL22, CCL17, CXCL12, TGF-Β, IL-12, miR-12/PEG3 and IRF-8; Secondly, TAMs promote tumor antigen recognition disorders via LINC01232/E2F2/NBR1/MHC-I and TPL2/Erk/CIITA; Moreover, TAMs regulate the secretion of immunosuppressive factors via IL-6, IL-10, TGF-Β, VEGF-A, and interfere with immune checkpoints via p65/STAT3-CSN5 and CD30L/CD30.

Tumor immune escape exerts key effects on unresponsive chemotherapy, immune therapy ineffective, poor prognosis, etc. The main ways of tumor cell immune escape are (1): tumor immunogenicity decreased; (2) the maturation of dendritic cells (DCs) was inhibited; (3) T lymphocytes activity was inhibited; (4) T lymphocytes migration and infiltration were inhibited; (5) the recognition function of immune cells was inhibited; (6) increased expression of immune checkpoint; (7) immunosuppressive cells were recruited (48). According to substantial evidences, TAMs play a pivotal role in tumor immune escape by regulating the recruitment and function of immune cells, promoting tumor antigen recognition disorders, regulating the secretion of immunosuppressive factors, and interfering with immune checkpoints via different mechanisms through various cytokines and pathways (**Figure 2**).

### 3.2 TAMs regulate recruitment and function of immune cells

T cells play a key role of immune response, they are mainly classified into cytotoxic T cells, helper T cells and regulatory T cells. Among them, CD8^+^ cytotoxic T cells (CTL) mainly recognize antigen peptides presented by major histocompatibility complex (MHC) class I molecules and directly kill tumor cells. Whereas CD4^+^ T helper cells usually recognize MHC class II antigens and indirectly help clear tumor cells by secreting cytokines such as IL-2 and IFN-γ that enhance phagocytes and other T cells; Tregs prevent overimmunity, inhibit the role of cytotoxic T cells in a variety of tumors, and are generally believed to be associated with tumor immune escape (49). It has been reported that Fas ligand (FasL) on TAMs would bind to Fas receptor on immune cells, subsequently promoting the expression of caspase 3 and caspase 8, further directly regulating immune cells apoptosis, therefore, TAMs exhibit immunosuppression function (50).

In TME, TAMs recruit immunosuppressive cells and facilitate the depletion of cytotoxic T cells, thereby serving as a mediator in promoting immune escape. The infiltration of M2-type TAMs and Tregs is increased in nasopharyngeal carcinoma, and high infiltration level is associated with poor prognosis. Mechanistically, nasopharyngeal carcinoma cells induced polarization of M2-type TAMs by secreting TGF-Β1 and IL-10. Subsequently, M2-type TAMs induced Tregs migration by secreting CCL22, CCL17, CXCL12, and fostered Tregs transformation by secreting TGF-Β and IL-2, which led to the recruitment of Tregs and the immune escape of nasopharyngeal carcinoma cells (51). In addition, using CD4^+^ Foxp3^+^ Treg and monocytes co-culture system, it was found that the expression of CD206 and CD163 increased through IL-10, IL-13 and IL-4 secreted by Tregs (52). Exosomes are endogenous extracellular vesicles with a diameter of 30-100 nm, they are the mediators of intercellular communication (53). As reported by Yang et al. (54), exosomes derived from M2-type TAMs increased microRNA-21 (miR-21) expression and decreased paternally expressed gene 3 (PEG3), subsequently increased the expression of Bcl-2, MMP-2 and MMP-9, reduced the proliferation and cytotoxic activity of CTL. It also enhanced Ki67 and PCNA expression, as well as the volume of tumors by downregulating the proportion of CTL, eventually inhibited the apoptosis of glioma cells, mediating immune escape. In addition, Nixon et al. (55) found that interferon regulatory factor-8 (IRF8) drives antigen presenting function of TAMs, induced the expression of PD-1 on CTL, thus leading to the depletion of CTL, weakening the immune response and forming an immunosuppressive environment. In summary, TAMs participate in tumor immune escape by regulating the recruitment and function of immune cells.

TAMs induce dysrecognition of tumor antigens. Tumor cells evade T cell recognition by removing, reducing, and transforming MHC-I molecules on their surface, resulting in tumor immune escape (48). It was found that the exosome LINC01232 antigen from M2-type TAMs promoted the transcription of NBR1 by binding to E2F2, and NBR1 binds to MHC-I protein, mediating the increased degradation of MHC-I in the autophagolysosome, eventually decreased the expression of MHC-I on the surface of tumor cells. In this way, tumor cells protected themselves from the immune attack of CTL, thus immune escape occurred (56). The surface of TAMs also express MHC-II, which presented tumor antigens to CD4^+^ T cells (57). Wu et al. (58) found that podoplanin (PDPN), a mucin-like transmembrane glycoprotein, could promote the polarization of M2-type TAMs through the secretion of exosomes. Additionally, PDPN could reduce the expression of MHC-II on the surface of macrophages through the tumor progression locus2/Erk/MHC class II transactivator (TPL2/Erk/CIITA) pathway and impair the antigen presentating ability. In turn, it affected the activity of CD4^+^ T cells and promoted the immune escape of glioblastoma. Therefore, TAMs induced tumor antigen recognition barriers by affecting MHC molecules on the surface of tumor cells, and promoted the occurrence of tumor immune escape.

### 3.3 TAMs regulate the secretion of immunosuppressive cytokines

Tumor immunosuppressive state would not have been possible by the recruitment of immunosuppressive cells in TME alone, but rather with the affect of immunosuppressive factors. IL-10 is a kind of immunosuppressive cytokine in TME promotes the polarization of M2-type TAMs, and in turn M2-type TAMs secrete more IL-10. A recent study have proved that microRNA let-7d targeted with IL-10 and inhibited the polarization of M2-type TAMs. That is, let-7d inhibited the crosstalk between M2-type TAMs and IL-10, thereby preventing immune escape in renal cell carcinoma (59). It is clear that IL-6 regulates macrophages, T cells and other immune cells, it has closely connection with tumor immunosuppressive microenvironment. Ma et al. (9) found that the exosome miR-1555p derived from TAMs promoted the secretion of IL-6 by down- egulating the expression of ZC3H12B in colon cancer cells both *in vivo* and *in vitro*. Subsequently, IL-6 affects the response of T cells, leading to immune escape of colon cancer and tumor progression. Similarly, in colorectal cancer, TAMs secreted the immunosuppressive factor TGF-Β, promoted the expression of hypoxia-inducible factor 1α (HIF-1α) and then increased tribbles pseudokinase 3 (TR1B3), activated the Β-catenin/Wnt signaling pathway, enhanced the expression of PD-L1 on the cell membrane surface, and played a role in cell invasion and tumor immunity (10). In addition, M2-type TAMs are also involved in chemotherapy resistance through the secretion of immunosuppressive cytokines. In the lung cancer mouse model, M2-type TAMs could accumulate in well-differentiated tumor vascular sites, and promote tumor vascular reconstruction and recurrence by secreting vascular endothelial growth factor-A (VEGF-A) (60). Therefore, TAMs modulate the secretion of immunosuppressive cytokines to promote tumor immune escape.

### 3.4 TAMs interfere with immune checkpoints

Immune checkpoint is one of the targets of immunotherapy, including PD-1/PD-L1, CTLA-4, etc. PD-1 expresses on the surface of immune cells such as CD8^+^ T cells and CD4^+^ T cells, while PD-L1 expresses on the surface of APCs such as tumor cells and TAMs. The binding of the PD-1 and PD-L1 inhibits the cytotoxicity of immune cells and mediates immune escape. Immune checkpoint inhibitors can block these immune checkpoint ligands, so that immune cell activity can be restored and immune escape can be suppressed. In recent years, accumulating evidences have shown that TAMs affected tumor immunity by interfering with the expression of immune checkpoints. Liu et al. (61) found that CC motif chemokine ligand 5 (CCL5) secreted by TAMs induced upregulation of PD-L1 protein in colon cancer cells through p65/signal transducer and activator of transcription 3/COP9 signalosome 5 (p65/STAT3-CSN5) pathway, which enabled colon cancer cells to evade immune surveillance. On the other hand, inhibition of CTL mediated cytotoxicity promotes cellular immune escape and ultimately accelerated the development of colon cancer. Wang et al. (62) found that CD30L/CD30 signal increased the expression of PD-L1 on the cell membrane of TAMs. Meanwhile, PD-L1 on TAMs increased its binding to PD-1 on the surface of effector CD8^+^ T cells and CD4^+^ T cells, so it weakened the immune attack of CD8^+^ T cells and CD4^+^ T cells, and then mediated immune escape of colon cancer. In addition, in gastric cancer, TAMs-derived exosomes also mediated immune escape of gastric cancer cells by promoting PD-L1 expression in cancer cells (63). Therefore, interference with the expression of immune checkpoints is also a crucial way for TAMs to participate in tumor immune escape.

## 4 STC1 regulates macrophage function

### 4.1 STC1 regulates macrophage polarization and subpopulation differentiation

The polarization of macrophages plays a crucial role in inflammatory response and tumor immunity. Studies have shown that STC1 was secreted by CAFs and suppressed macrophage differentiation by binding to glucose-regulated protein 94 (GRP94), suggesting that STC1 exhibited an essential influence on tumor macrophages differentiation and participated in tumor immunity (64). Human acute monocytic leukemia THP-1 cells are generally used to induce M0, M1 and M2 macrophages. To explore the relationship between STC1 and TAMs differentiation, Leung et al. (65) induced THP-1 cells into M0 cells via phorbol 12-myristate 13-acetate (PMA), further induced M0 into M1-type macrophages via LPS/IFN-γ, and induced M0 into M2-type macrophages via IL-4/IL-3. It was found that the expression of STC1 increased in differentiated M0, M1 and M2 macrophages, among them, the level of STC1 is highest in M1-type macrophages. Additionally, using siRNA_STC1_ interference, this study investigated the effect of downregulation of STC1 gene in macrophages on the migration of hepatocellular carcinoma cells Hep3B. The results showed that the decrease of STC1 expression promoted the expression of TBC1 domain family member 3 (TBC1D3), led to the decrease of STAT phosphorylation level, and further inhibited the proliferation and migration of hepatocellular carcinoma *in vitro*. Different from previous studies, M1-type macrophages tend to have pro-inflammatory effects, while STC1 tends to have anti-inflammatory effects. This paradoxical effect in this study may be due to the fact that STC1 negatively regulated the inflammatory response as an endogenous anti-inflammatory mediator and avoided the continuous activation of immune cells. In the myocardial infarction mouse model, Arezoo et al. (66) explored the impact of STC1 on the differentiation of monocytes/macrophages. The release of stimuli such as pathogen-associated molecular patterns (PAMPs), damage-associated molecular patterns (DAMPs) and chemokines was elevated due to tissue injuring, these stimulants promoted the differentiation of monocytes and migration to the injured tissues. The addition of rhSTC1 decreased the expression of CD14 and the response to inflammatory stimuli, thus alleviated the continuous detrimental inflammatory response. A recent study found that STC1 promoted the immunosuppressive microenvironment by facilitating the polarization and infiltration of M2-type TAMs. By mechanism, STC1 activated Yes-associated protein (YAP) and promoted the secretion of CCL2, which induced the polarization of M2-type TAMs. Subsequently, M2-type TAMs secreted VEGF-A, which in turn increased the expression of STC1 by Akt signaling pathway. And the increase of YAP brought higher expression of PD-L1 on the melanoma cells, which decrease the interaction between macrophages and tumor cells (67). Taken together, these studies confirmed that STC1 served a role in the differentiation of macrophage subsets. It was also able to influence tumor proliferation and migration, but the role of STC1 in macrophage subpopulation differentiation needs more researches to confirm.

### 4.2 STC1 regulates macrophage activation and inflammatory response

Macrophages participate in the inflammatory response through the production of superoxide, inflammasome Nod-like receptor protein 3 (NLRP3), Nod-likenreceptor containing a caspase activating and recruitment domain 4 (NLRC4), IL-1Β and other pathways, and studies have shown that STC1 can modulate these processes. The function of macrophages is affected by mitochondrial superoxide, and uncoupling protein 2 (UCP2) plays a major role in this process. Studies have found that STC1 can be internalized by macrophages and localized in mitochondria within 10 minutes, and STC1 induced the expression of UCP2 in macrophages, thereby weakening the influence of LPS on the generation of superoxide in macrophages. In general, STC1 exerted an impact on macrophage inflammation and immune response by regulating the generation of superoxide in macrophages (68). Mesenchymal stem cells (MSCs) are a class of stem cells with multi-differentiation potential and self-replication ability. JOO YOUN OH et al. (69) found that hMSCs derived from bone marrow reduced mitochondrial reactive oxygen species (ROS) of macrophages by secreting STC1, thus inhibited the activation of macrophage inflammasome NLRP3. What’s more, STC1 secreted by adipose-derived mesenchymal stem cells (ASCs) inhibited inflammasome NLRC4, alleviated the activation of caspase-1 and the secretion of IL-1Β and IL-18, thus regulated the inflammation response of macrophages (70). Similarly, Yoojin Seo et al. (71) found that the expression level of STC1 in tonsillar derived MSCs (TMSCs) was higher than that of other MSCs. Inhibition of STC1 could completely eliminate the influence of TMSCs on ROS, and further weaken the inhibition effect of TMSCs on IL-1Β production in macrophages. These results have established that STC1 regulated the anti-inflammatory effect of macrophages through ROS pathway (71). To elucidate the mechanism of alveolar macrophages in acute respiratory distress syndrome, Xia et al. (72) co-cultured LPS-stimulated alveolar macrophages with human umbilical mesenchymal stem cells (HUMSC), it is found that the level of STC1, PI3K, AKT and mTOR increased, and IL-10 secreted by macrophages also elevated. Then, by knocking down STC1 gene and inhibiting mTOR with rapamycin, it was demonstrated that HUMSC secreted STC1, regulated the secretion of IL-10 by alveolar macrophages through PI3K/AKT/mTOR pathway. In conclusion, STC1 affects the inflammatory response of macrophages.

### 4.3 STC1 affects the phagocytosis activity and antigen presentation of macrophages

In 2004, researchers discovered that STC1 mapped to macrophages, it also reduced intracellular calcium levels, inhibited macrophages binding with chemotactic protein-1 (MCP-1) and stromal cell derived factor-1α (SDF-1α) on the monocytes, thereby affected the chemotaxis and response of a macrophages to antigens (13). Besides, endothelium greatly influenced the migration of macrophages from the circulation to impaired tissues, STC1 kept the tight junctions among endothelial cells by blocking TNF-α or IL-1Β on the cells, ultimately inhibited the transendothelial migration of macrophages (73, 74). In other words, STC1 was capable of inducing hypofunction or defect of macrophages such as migration and response to antigens, thus leading to tumor immune escape. In 2021, STC1 was reported to be a phagocytic checkpoint. Specifically, STC1 decreased the “eat me” signal by down-regulating the expression of CRT on the membrane of cancer cells, affecting the recognition of cancer cells by antigen presenting cells such as TAMs and DCs, and further interfered with the specific recognition of T cells. That is, STC1 can drive the function of macrophages antigen presentation by influencing phagocytosis signals (75). Ferroptosis tends to occur in the injured endomentosis, large peritoneal macrophages (LPMs) will migrate to the uteri to against ferroptotic amonocytes/macrophages, which promotes anti-inflammatory response. It has been reported that MSCs facilitated the efferocytosis of LPMs, and STC1 showed an essential function in this process. By using rhSTC1 and siRNASTC1, Wang et al. (76) found MSCs-derived STC1 promoted the migration of LPMs to injured uteri, further accelerated the phagocytosis activity of LPMs. Collectively, STC1 can affect the phagocytic activity and antigen presentation of TAMs.

## 5 Discussion

**Figure 3:**
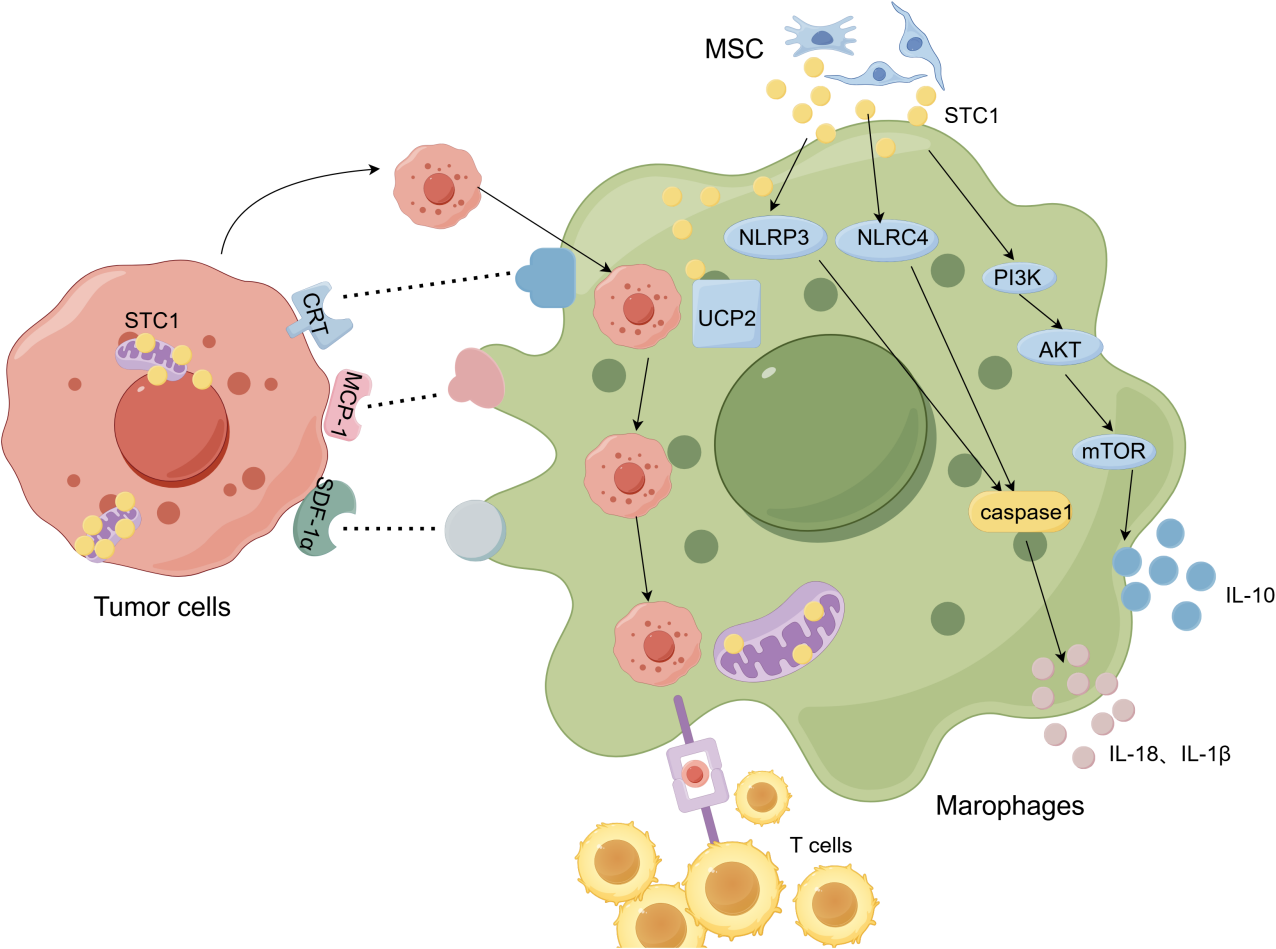
The relationship between Stanniocalcin-1 (STC-1) and macrophages. (By Figdraw.). STC1 is secreted by MSCs and targeting to the inner mitochondrial membrane and tumor cells also express STC1. The proteins such as CRT, MCP-1, SDF-1α on the tumor cells binds to ligands on the TAMs, thus affect antigen presenting function and phagocytosis of TAMs. STC1 promotes PI3K/Akt/mTOR pathway and subsequently facilitates TAMs secreting IL-10. What’s more, STC1 have influences on TAMs via inflammasome NLRP3, NLRC4 and UCP2.

TAMs play a critical role in tumor immunity and exert various influences on the proliferation, migration, invasion and metastasis of cancer cells. STC1 has been widely studied as a phagocytosis checkpoint in recent years. From various researches, it has been proved that STC1 makes differences on the infiltration and function of different aspects of macrophages (**Figure 3**). However, in TME, how STC1 affects the functions of TAMs, such as whether STC1 reshapes the macrophage by metabolic pathway, glucose, lipid or amino acid, it still needs more experiments to explore. To be certain, current studies have confirmed that STC1 has a pro-cancer effect, so the design and development of STC1-targeted drugs are crucial for immunotherapy. There are currently no clinical trials of drugs targeting STC1, but some preclinical studies have found that targeting STC1 can slow tumor progression. For example, STC1 had been proved as a target gene for miR-146b-5p (77), and sevoflurane could upregulate the expression of miR-146b-5p and downregulate the expression of STC1 to suppress tumor growth (78); Choi et al. (79) found STC1 was the target gene of miR-606 by using the miRNA target prediction program and miR-606 mimics transfection, and it inhibited tumor growth and metastasis *in vivo*. In this way, the possible directions of STC1 in tumor therapy are as follows (1): Elucidate the three-dimensional structure of STC1, identify its receptor, and conduct a comprehensive study on its downstream signaling pathway; (2) Clarify the prediction value of STC1 in different tumors through multiple clinical studies, and explore the application of STC1 gene detection serve as a biomarker for tumor diagnosis, prevention and treatment; (3) Develop monoclonal antibodies against STC1 to block the action of STC1 and inhibit immune escape; (4) Develop small molecule compounds that mimic STC1 siRNA to reduce the expression level of STC1 *in vivo*, thereby inhibiting tumor. In addition, based on the effects of STC1 on TAMs, drugs that inhibit STC1 and TAMs, block the crosstalk between STC1 and TAMs should be an underlying direction of immunotherapy. Meanwhile, combination of STC1 inhibitor and TAMs inhibitor can be studied to achieve better immunotherapy effects. However, based on the regulation of calcium and phosphorus by STC1, safety problems targeting STC1 should be considered, and further researches are required to insure above approaches can bring clinical benefits for cancer.

In summary, tumor immunotherapy has manifested unprecedented success for cancer patients by enhancing the body immunity level to achieve the objective of killing tumor cells, whereas in clinical practice, only 20%-40% of patients can benefit from immunotherapy (2), “immune escape” phenomenon is considered to be the crucial reason for the failure of immunotherapy. Specifically, the reasons of tumor immune escape contains reducing the autoimmunogenicity, inhibiting the maturation and infiltration of immune cells, destroying the recognition function of immune cells, recruiting immunosuppressive cells, and interfering with immune checkpoints. STC1 is a glycoprotein hormone associated with metabolism of calcium and phosphorus, and a large number of studies have elucidated that STC1 is involved in the occurrence and development of tumors. It was demonstrated that STC1 could act by influencing TME, participating in EMT and interfering with phagocytosis signals. Additionally, TAMs also show its ability in tumor immune escape by promoting tumor antigen recognition disorder, regulating the recruitment and function of immune cells, regulating the secretion of immunosuppressive factors, and interfering with immune checkpoints. In 2021, STC1 was shown to act as a phagocytic checkpoint and a TAMs function regulator, as well as participate in tumor immune escape, suggesting the interaction between STC1 and TAMs. In the future, more studies are needed to explore the relationship between STC1 and TAMs, and develop corresponding drugs to optimize the therapeutic effect of tumors. With the further studies on the crosstalk between STC1 and TAMs in the TME, may have the potential to makes STC1 to become a novel biomarker, which can improve the accuracy of early diagnosis of tumors and determine tumor metastasis. Hence, these strategies will help achieve a better anti-tumor potency of immunotherapy.

## Glossary

PD-1: programmed death-1 receptor
PD-L1: programmed death-1 receptor ligand 1
CTLA-4: cytotoxic T lymphocyte-associated antigen-4
IDO: indoleamine-2,3-dioxygenase
TME: tumor microenvironment
TAMs: tumor-associated macrophages
NK cells: natural killer cells
Tregs: regulatory T cells
STC1: Stanniocalcin-1
EMT: epithelial-mesenchymal transition
Notch1-SOX2: notch1-sex determining region Y-box 2
ERK1/2: extracellular signalregulatedkinases1/2
NF-ΚB: Nuclear Factor-kappaB
JNK: and Jun-N-terminal kinase
TNBC: triple negative breast cancer
HER2^+^: human epidermal growth factor receptor 2^+^
CAFs: cancer associated fibroblasts
NFs: normal fibroblasts
rhSTC1: human recombinant STC1
STC1 Ab: monoclonal antibody STC1
APC: antigen presenting cells
CRT: calreticulin
PD1A3: protein disulfide isomerase associated 3
IP: immunoprecipitation
CAR-T: chimeric antigen receptor T-cell immunotherapy
hMSCs: human mesenchymal stem cells
LPS: lipopolysaccharide
TNF-α: tumor necrosis factor-α
IFN-γ: interferon-gamma
CCL: CC motif chemokine ligand
CXCL: C-X-C motif chemokine ligand
DCs: dendritic cells
CTL: CD8^+^ cytotoxic T cells
MHC: major histocompatibility complex
FasL: Fas ligand
TGF-Β: transforming growth factor-beta
miR-21/PEG3: microRNA-21/paternally expressed gene 3
IRF8: interferon regulatory factor-8
PDPN: podoplanin
TPL2/Erk/CIITA: tumor progression locus2/Erk/MHC class II transactivator
HIF-1α: hypoxia-inducible factor 1α
TR1B3: tribbles pseudokinase 3
VEGF-A: vascular endothelial growth factor-A
p65/STAT3-CSN5: p65/Signal transducer and activator of Transcription 3/COP9 signalosome 5
GRP94: glucose-regulated protein 94
PMA: phorbol 12-myristate 13-acetate
TBC1D3: TBC1 domain family member 3
PAMPs: pathogen-associated molecular patterns
DAMPs: damage-associated molecular patterns
YAP: Yes-associated protein
NLRP3: Nod-like receptor protein 3
NLRC4: Nod-liken receptor containing a caspase activating and recruitment domain 4
UCP2: uncoupling protein 2
MSCs: mesenchymal stem cells
ROS: reactive oxygen species
ASCs: adipose-derived mesenchymal stem cells
TMSCs: tonsillar derived MSCs
HUMSC: human umbilical mesenchymal stem cells
MCP-1: chemotactic protein-1
SDF-1α: stromal cellderived factor-1α
LPMs: large peritoneal macrophages.
